# Identification and validation of genes associated with mitochondria in cerebral infarction and benign prostatic hyperplasia

**DOI:** 10.3389/fmed.2026.1964927

**Published:** 2026-09-22

**Authors:** Hongjian Tu, Shanzhi Zhan, Leihua Cao, Yao Wang, Hongwei Yuan, Xiangyun Xu, Yumei Li

**Affiliations:** 1 Department of Urology, Nanchang People’s Hospital, Nanchang, China; 2 Department of Neurology, Nanchang People’s Hospital, Nanchang, China

**Keywords:** benign prostatic hyperplasia, cerebral infarction, machine learning, mitochondria, nomogram

## Abstract

**Background:**

Cerebral infarction (CI) and benign prostatic hyperplasia (BPH) are prevalent conditions in middle-aged and elderly individuals. Previous studies have demonstrated that mitochondrial dysfunction plays a critical role in both CI and BPH. This study aims to identify the mitochondria-related genes (MRGs) in CI and BPH, providing theoretical clues and preliminary candidate gene resources for further research on mitochondria targeted therapeutic strategies across different diseases.

**Methods:**

The transcriptomic data of BPH and CI were obtained from public databases, while MRGs were derived from published literature. Firstly, the differentially expressed genes (DEGs) in the combined and batch-effect-removed datasets were intersected with MRGs to obtain candidate genes, and their functions were explored. Then, hub genes were further screened through machine learning. Subsequently, the expression levels of hub genes, their predictive abilities for diseases, and the changes in the immune microenvironment were respectively analyzed in the relevant datasets of BPH and CI. Finally, RT-qPCR was used to verify the expression of hub genes in humans.

**Results:**

Eleven candidate mitochondrial genes were identified. They likely mediated CI and BPH pathogenesis via metabolic and cellular regulation. Further analysis showed that HSDL2 and PDK4 were downregulated in BPH, enabling effective BPH-control discrimination. The nomogram constructed based on these two genes also had a strong predictive ability for the risk of developing BPH. CYP1B1, GYG1, HSDL2, MXD1, SLC22A4, and TNFSF10 were significantly upregulated at high levels in the disease group and had a strong ability to distinguish between CI and control samples. The nomogram constructed based on these six genes showed a strong predictive ability for the risk of developing CI. In addition, activated B cells and neutrophils showed opposite infiltration trends in BPH and CI relative to healthy controls. Finally, RT-qPCR confirmed that the expression of HSDL2 and PDK4 in patients with comorbid CI and BPH (CIwPH) was significantly lower than that in normal controls.

**Conclusion:**

This study identified 8 hub genes and explored their application value in clinical identification, providing new research directions for the diagnosis and treatment of BPH and CI.

## 1 Introduction

Cerebral infarction (CI) is an acute cerebrovascular disorder caused by thrombotic or embolic vascular occlusion that interrupts cerebral oxygen and nutrient supply. Persistent neuroinflammation and immune-cell infiltration drive disease progression and unfavorable outcomes, particularly among middle-aged and elderly populations (1). Treatments for CI span acute vascular recanalization to long-term rehabilitation. Although intravenous thrombolysis and endovascular thrombectomy have improved acute-phase management, clinical applicability is constrained by time windows, technical barriers, and patient eligibility (2, 3).

Benign prostatic hyperplasia (BPH) consists of epithelial and fibromuscular proliferation within prostatic transition and peripheral zones, producing lower-urinary-tract symptoms including frequency, weak stream, hesitancy, and nocturia. Prevalence rises sharply with age in older men. Management is stratified according to symptom severity (IPSS score), quality-of-life impact, prostate volume, complications (urinary retention, hydronephrosis, recurrent infection), and patient preference (4–6). Transurethral resection of the prostate (TURP) remains the primary surgical modality, yet disease recurrence is possible. Notably, CI is a rare but serious postoperative complication of TURP. Importantly, CI and BPH frequently co-occur in aging men, and their common molecular mechanisms remain incompletely characterized (7, 8). Identification of shared molecular targets therefore carries substantial clinical relevance.

Mitochondria execute oxidative phosphorylation for ATP production and additionally govern calcium homeostasis, reactive oxygen species (ROS) homeostasis, and apoptotic signaling (9). Mitochondrial dysfunction is central to multiple degenerative diseases. In CI, ischemia-reperfusion injury collapses mitochondrial membrane potential, depletes ATP, releases cytochrome C, and activates caspase-dependent neuronal apoptosis (10). In BPH, disturbed mitochondrial dynamics (abnormal fission-fusion balance) is associated with stromal-cell overproliferation (11, 12). Whether CI and BPH share overlapping mitochondrial regulatory networks has not been systematically investigated (13). Individual studies have examined mitochondrial-related genes in CI or BPH separately, but cross-disease common regulatory modules remain under-explored.

Given global population aging, comorbid CI and BPH are increasingly prevalent. Current therapeutic strategies largely target single diseases, while shared pathological pathways remain underexplored. Herein, we combined multiple transcriptomic datasets, bioinformatic workflows, and MRG sets to identify common MRGs for CI and BPH. We constructed nomogram prediction models, evaluated immune-cell infiltration, and performed RT-qPCR validation in clinical samples. Elucidating shared mitochondrial-gene interactions provides new perspectives on how mitochondrial dysfunction contributes to both degenerative and proliferative pathologies in multimorbid elderly patients.

## 2 Materials and methods

### 2.1 Data collection

All datasets were downloaded from the Gene Expression Omnibus (GEO; https://www.ncbi.nlm.nih.gov/geo) (14). BPH training datasets: GSE7307 (GPL570) was used as a training dataset and included 7 BPH prostate tissue samples (GSM175918–GSM175922, GSM175929, and GSM175932) and 12 control prostate tissue samples (GSM175923–GSM175928, GSM175955, GSM176277, GSM176278, and GSM176325–GSM176327). GSE119195 (GPL6244), also used for training, included 5 BPH samples (GSM3360636–GSM3360640) and 3 control samples (GSM3360641–GSM3360643). The independent BPH validation dataset GSE132714 (GPL16791) comprised 18 BPH samples (GSM3890639–GSM3890656) and 4 control samples (GSM3890657–GSM3890660). For CI, the training dataset GSE16561 (GPL6883) comprised 39 peripheral blood samples from patients with acute CI (GSM416528–GSM416566) and 24 healthy control samples (GSM416567–GSM416590). GSE102541 (GPL22755), also used for training, included 6 peripheral blood samples from patients with acute CI (GSM2740405–GSM2740410) and 3 healthy control samples (GSM2740411–GSM2740413). The independent CI validation dataset GSE58294 (GPL570) included 69 peripheral blood samples from patients with acute CI (GSM1406056–GSM1406124) and 23 healthy control samples (GSM1406033–GSM1406055). A total of 2,030 MRGs were extracted from published literature (15) (Supplementary Table 1).

### 2.2 Data processing and identification of differentially expressed genes (DEGs)

Training-set raw data were imported using the R package GEOquery (v2.38.4) (16). Batch-effect correction was performed with sva (v3.46.0) (17), and boxplots visualized normalization outcomes. The limma (v3.54.0) package (18) identified DEGs for BPH and CI with thresholds |log₂FC| > 0.5 and *P* < 0.05. Volcano plots and heatmaps of top-ranked DEGs were rendered using ComplexHeatmap (v2.14.0) (19).

### 2.3 Identification and functional enrichment analysis of candidate genes

Common-DEGs between BPH-DEGs and CI-DEGs were obtained via ggvenn (v0.1.9) (20). Candidate genes were defined as the intersection between Common-DEGs and MRGs. clusterProfiler (v4.8.3) (21) performed GO (adjusted *P* < 0.05) and KEGG (*P* < 0.05) enrichment analyses. ggplot2 (v3.4.1) (22) visualized top-5 GO terms (BP, CC, MF) and top-10 KEGG pathways.

### 2.4 Machine-learning and expression analysis

Least absolute shrinkage and selection operator (LASSO) regression was implemented via glmnet (v4.1.4) (23) using candidate genes; feature genes were selected at lambda.min. Overlap between BPH-LASSO-genes and CI-LASSO-genes defined hub genes. Wilcoxon rank-sum tests compared gene expression across groups in training and validation sets. Biomarkers required consistent expression trends and significant inter-group differences (*P* < 0.05) in both training and validation cohorts.

### 2.5 Performance evaluation of biomarkers

Receiver-operating-characteristic (ROC) curves were generated with pROC (v1.18.0) (24). An AUC > 0.7 was interpreted as strong discriminatory ability.

### 2.6 Construction and evaluation of the nomogram

Nomogram models were built on biomarker expression values using rms (v6.5.0) (25) for BPH and CI training datasets. Calibration curves were computed via ResourceSelection (v0.3.6); mean absolute error (MAE) < 0.1 indicated high predictive accuracy.

### 2.7 Immune-infiltration analysis

Single-sample gene-set enrichment analysis (ssGSEA) within GSVA (v1.46.0) (26) calculated enrichment scores for 28 immune-cell types. Wilcoxon tests identified immune-cell populations differing between disease and control groups (*P* < 0.05). psych (v2.1.6) (27) computed Spearman correlations between hub-gene expression and differentially infiltrated immune cells; |cor| > 0.3 and *P* < 0.05 were considered significant. However, ssGSEA provides transcriptome-derived enrichment scores for immune-cell signatures rather than absolute cell counts; it serves as a computational estimation and cannot replace flow cytometry or immunohistochemistry for direct immune-cell quantification.

### 2.8 Real-time quantitative reverse-transcription PCR (RT-qPCR)

Total RNA was isolated from clinical tissue samples. In the CIwPH group and the control group, 5 individuals were included each. The age range of the CIwPH group was from 65 to 72 years old, while that of the control group was from 63 to 75 years old. Reverse transcription was performed using Hifair® III 1st Strand cDNA Synthesis Supermix for qPCR (Yeasen, Shanghai, China). RT-qPCR was run on a Bio-Rad CFX96 instrument with SYBR-Green chemistry. Primer sequences: - HSDL2: forward 5′-CATGTTACCCAACACCGGGA-3′; reverse 5′-TGCTCCATCCTTTGCTGCTT-3′ - PDK4: forward 5′-ATGGTGGCCTAGTGTTGTGG-3′; reverse 5′-AATGCATGAGCTGGACTCCC-3′ - GAPDH: forward 5′-ATGGGCAGCCGTTAGGAAAG-3′; reverse 5′-AGGAAAAGCATCACCCGGAG-3′

Relative mRNA abundance was quantified by the 2⁻^*ΔΔ*^Ct method.

### 2.9 Statistical analysis

All bioinformatic analyses were performed in R 4.3.1. Group-wise comparisons used Wilcoxon rank-sum tests. Statistical significance threshold was *P* < 0.05.

## 3 Results

### 3.1 Identification of common-DEGs for BPH and CI

After batch-effect removal, sample-expression distributions were well-normalized (Figure 1A, Supplementary Figure 1). In BPH datasets, 2,018 BPH-DEGs were detected (159 upregulated, 1,859 downregulated; *P* < 0.05, |log₂FC| > 0.5; Figures 1B-C; Supplementary Table 2). For CI, 404 CI-DEGs were obtained (297 upregulated, 107 downregulated; Figures 1D-E; Supplementary Table 3). The intersection yielded 7,647 Common-DEGs shared by BPH and CI (Figure 1F).

**Figure 1:**
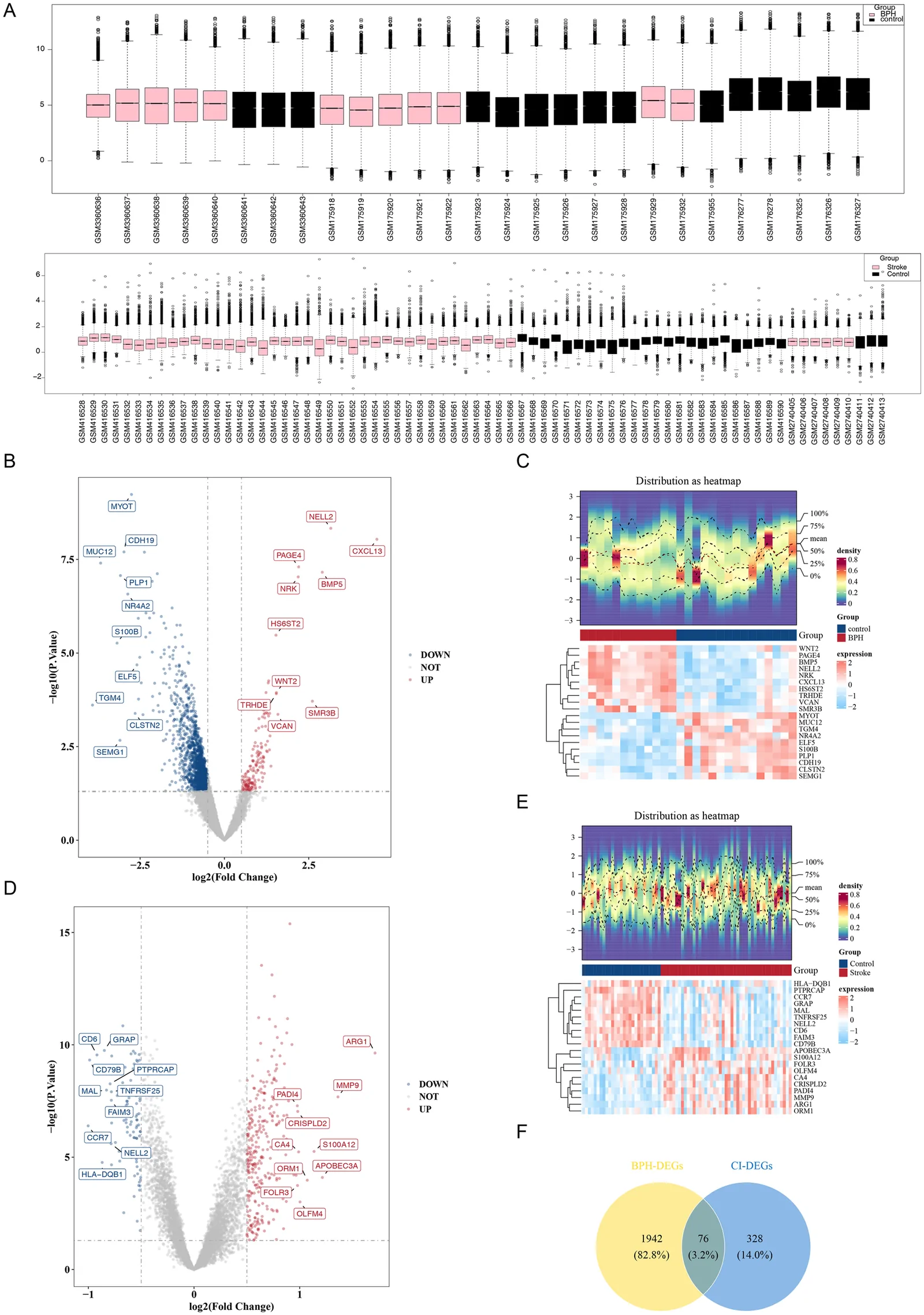
Identification of differentially expressed genes (DEGs) in BPH and CI datasets. **(A)** Boxplots illustrating expression-value distributions after batch-effect correction for CI and BPH training sets. **(B)** Volcano plot of DEGs comparing BPH versus control samples. **(C)** Heatmap of top-ranked BPH-related DEGs. **(D)** Volcano plot of DEGs comparing CI versus control samples. **(E)** Heatmap of top-ranked CI-related DEGs. **(F)** Venn diagram showing overlap between BPH-DEGs and CI-DEGs, yielding 7,647 Common-DEGs.

### 3.2 Functional enrichment of candidate genes

Intersection of Common-DEGs and MRGs identified 11 candidate genes (Figure 2A). Candidate genes were enriched in 102 GO terms (65 MF, 8 CC, 29 BP; adjusted *P* < 0.05; Supplementary Table 4). Top enriched terms included extrinsic apoptotic signaling pathway, fatty-acid metabolic process, peroxisome, microbody, and glycogen glucosyltransferase activity (Figure 2B), pointing to roles in apoptosis, metabolic regulation, and oxidative stress. KEGG enrichment returned 29 significant pathways including fatty-acid biosynthesis, tryptophan metabolism, and terpenoid biosynthesis (*P* < 0.05; Figure 2C; Supplementary Table 5).

**Figure 2:**
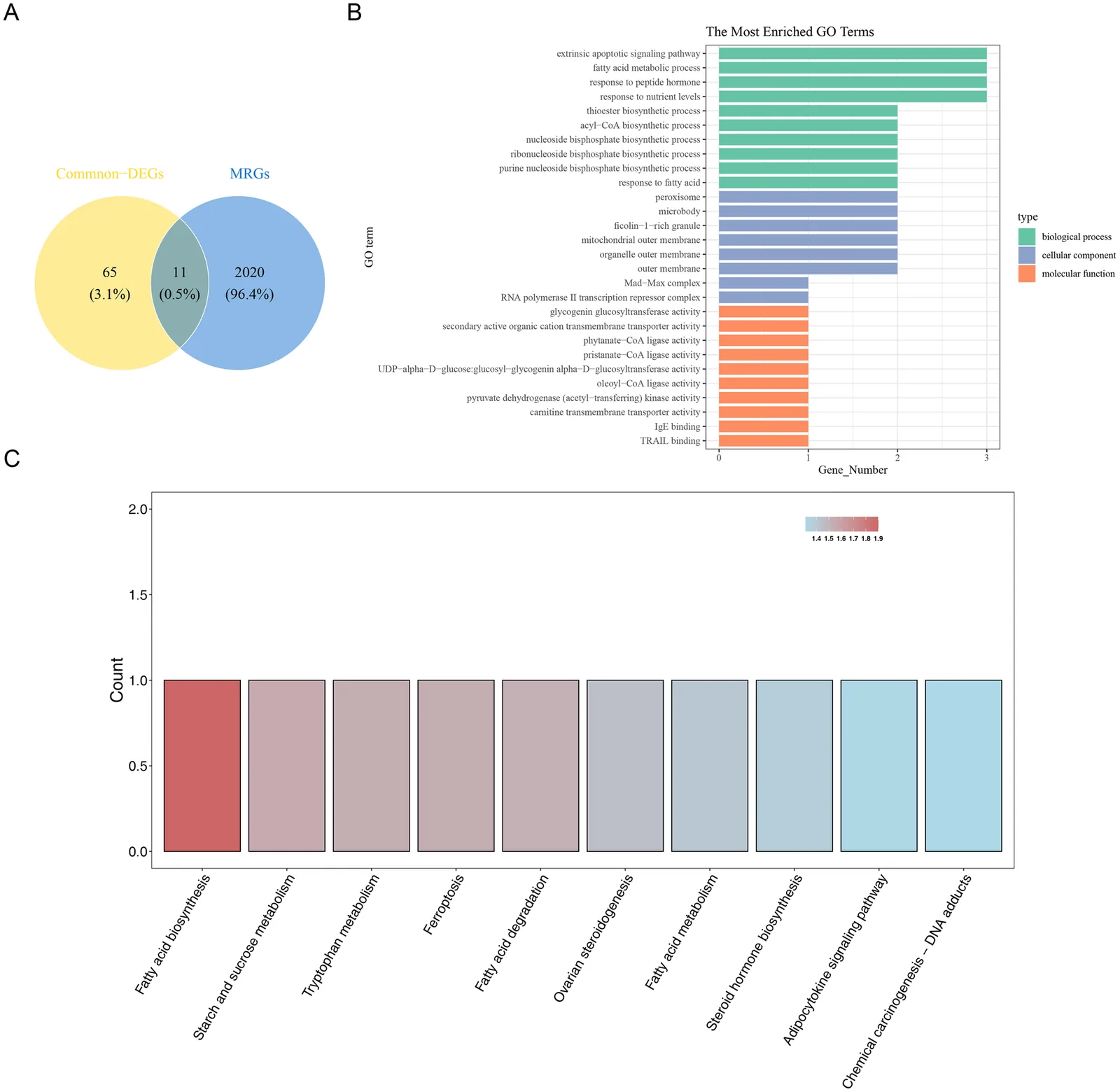
Functional enrichment for candidate genes overlapping with MRGs. **(A)** Venn diagram displaying intersection of Common-DEGs and MRGs, yielding 11 candidate genes. **(B)** GO enrichment dot plot for top-5 significant terms in biological process (BP), cellular component (CC), and molecular function (MF) categories (adjusted *P* < 0.05). **(C)** KEGG pathway enrichment for candidate genes (*P* < 0.05). See Supplementary Tables 2, 3 for full enrichment outputs.

### 3.3 Identification of eight hub biomarkers

LASSO regression selected 10 BPH-associated genes at lambda.min = −4.6826 (Figure 3A) and eight CI-associated genes at lambda.min = −2.915 (Figure 3B). Their intersection produced eight hub genes: CYP1B1, GYG1, IFI27, MXD1, SLC22A4, TNFSF10, HSDL2, PDK4 (Figure 3C). HSDL2 and PDK4 were consistently and significantly downregulated in BPH in both training and validation sets (*P* < 0.05; Figures 3D-E). In CI datasets, CYP1B1, GYG1, HSDL2, MXD1, SLC22A4, and TNFSF10 were significantly upregulated in disease samples (*P* < 0.001; Figures 3F-G).

**Figure 3:**
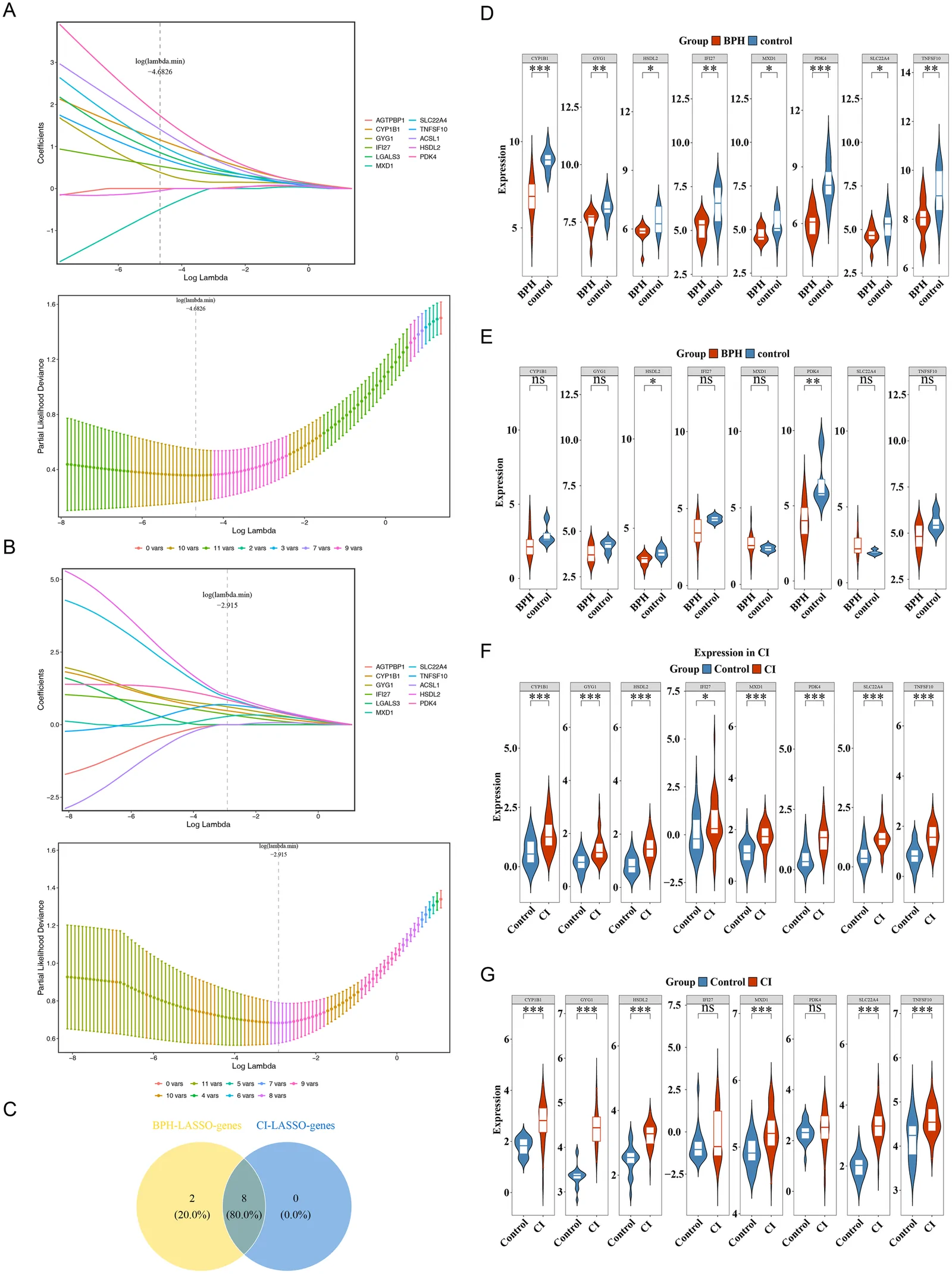
Machine-learning identification of mitochondrial-related hub biomarkers. (A-B) LASSO coefficient profiles for feature-gene selection. **(A)** BPH-associated genes; lambda.min = −4.6826 yielded 10 genes. **(B)** CI-associated genes; lambda.min = −2.915 yielded eight genes. **(C)** Venn diagram showing intersection of LASSO-selected genes from BPH and CI datasets, producing eight hub genes: CYP1B1, GYG1, IFI27, MXD1, SLC22A4, TNFSF10, HSDL2, PDK4. (D-E) Expression of HSDL2 and PDK4 in BPH training **(D)** and validation **(E)** datasets; both genes are significantly downregulated in BPH (*P* < 0.05). (F-G) Expression of six CI-related hub genes in CI training **(F)** and validation **(G)** datasets; genes are significantly upregulated in CI (*P* < 0.001). *P < 0.05, **P < 0.01, ***P < 0.001; ns = not significant vs. control.

### 3.4 Diagnostic performance and nomogram models

In BPH training data, AUC values were HSDL2 = 0.717 and PDK4 = 0.961 (Figure 4A); validation-set AUCs were HSDL2 = 0.875 and PDK4 = 0.931 (Figure 4B). For CI, all six biomarkers achieved AUC > 0.7 in training and validation cohorts (Figures 4C-D). Nomograms demonstrated that higher biomarker scores corresponded to greater disease risk (Figures 4E-F). Calibration-curve slopes approximated 1, indicating good predictive accuracy (Figures 4G-H). For BPH, PDK4 contributed most strongly; total score 135 corresponded to 89.3% BPH risk. For CI, TNFSF10 provided the largest contribution; total score 210 corresponded to 0.026% CI risk.

**Figure 4:**
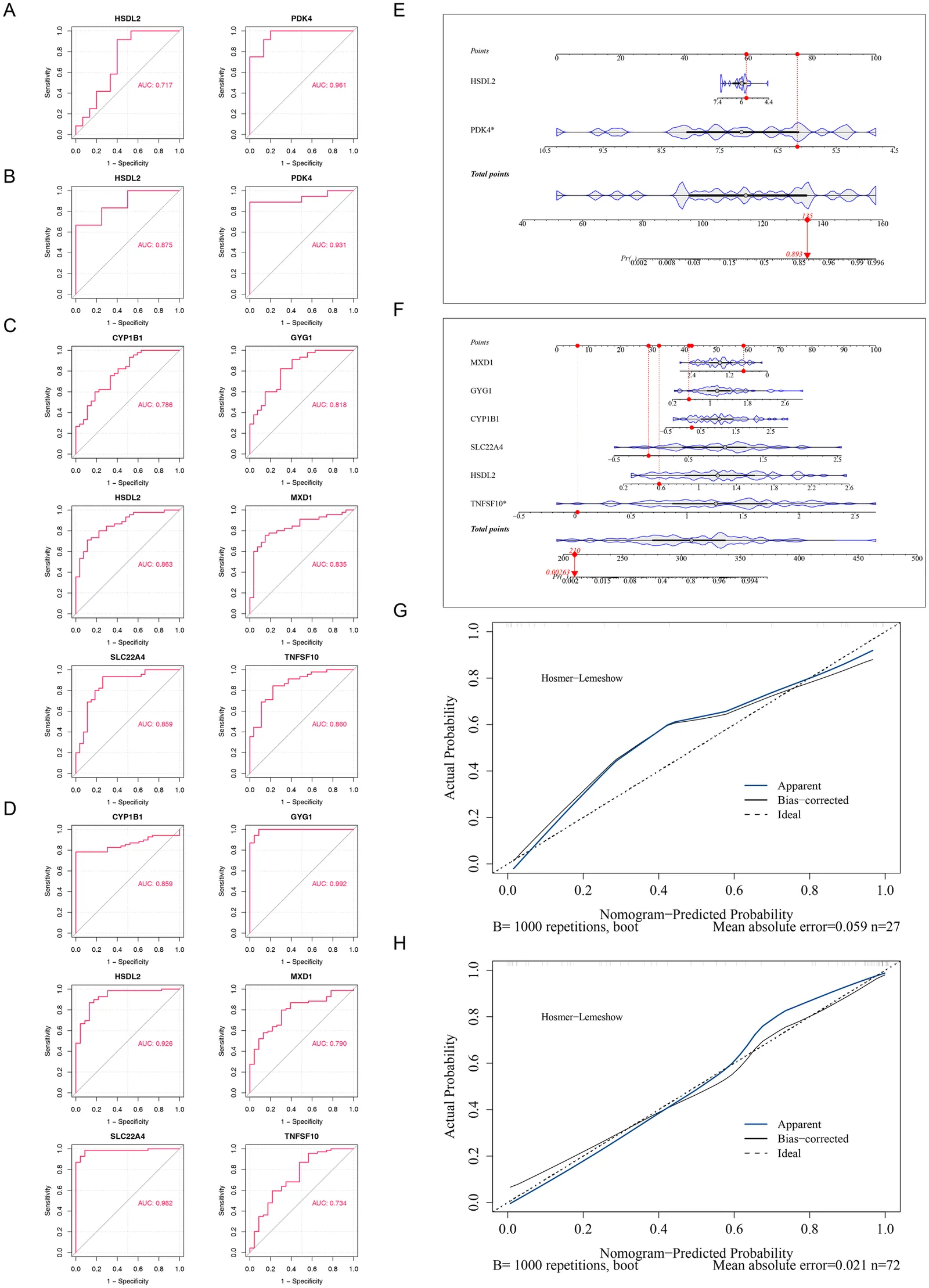
Diagnostic performance and nomogram-risk models for mitochondrial-related biomarkers. (A-B) ROC curves for BPH biomarkers. **(A)** Training-set AUCs: HSDL2 = 0.717, PDK4 = 0.961. **(B)** Validation-set AUCs: HSDL2 = 0.875, PDK4 = 0.931. (C-D) ROC curves for six CI biomarkers in training **(C)** and validation **(D)** datasets (all AUC > 0.7). (E-F) Nomogram risk-prediction models. **(E)** BPH nomogram built on HSDL2 and PDK4. PDK4 contributes most strongly; total score 135 corresponds to 89.3% BPH risk. **(F)** CI nomogram built on CYP1B1, GYG1, HSDL2, MXD1, SLC22A4, TNFSF10. TNFSF10 contributes most strongly; total score 210 corresponds to 0.026% CI risk. (G-H) Calibration curves for BPH **(G)** and CI **(H)** nomograms; slopes close to 1 indicate high predictive accuracy.

### 3.5 Remodeled immune microenvironment in BPH and CI

Eleven immune-cell populations differed significantly between BPH and control tissues (*P* < 0.05). Neutrophil infiltration was higher in controls; 10 other immune-cell types were enriched in BPH (Figure 5A). Seventeen immune-cell signatures differed in CI peripheral blood (*P* < 0.05). Activated B cells, activated CD4⁺ T cells, effector-memory CD8⁺ T cells, and activated CD8⁺ T cells were decreased in CI; 13 immune-cell populations were increased (Figure 5B). Notably, activated B cells and neutrophils exhibited opposite infiltration directions in BPH vs. CI relative to controls. Correlation analysis revealed that seven of eight hub genes (all except HSDL2) were positively correlated with neutrophils (|cor| > 0.3, *P* < 0.05). In BPH, PDK4 correlated negatively with activated dendritic cells (cor = −0.52, *P* < 0.05). In CI, all six CI biomarkers showed positive correlations with activated dendritic cells (cor > 0.4, *P* < 0.05; Figures 5C-D).

**Figure 5:**
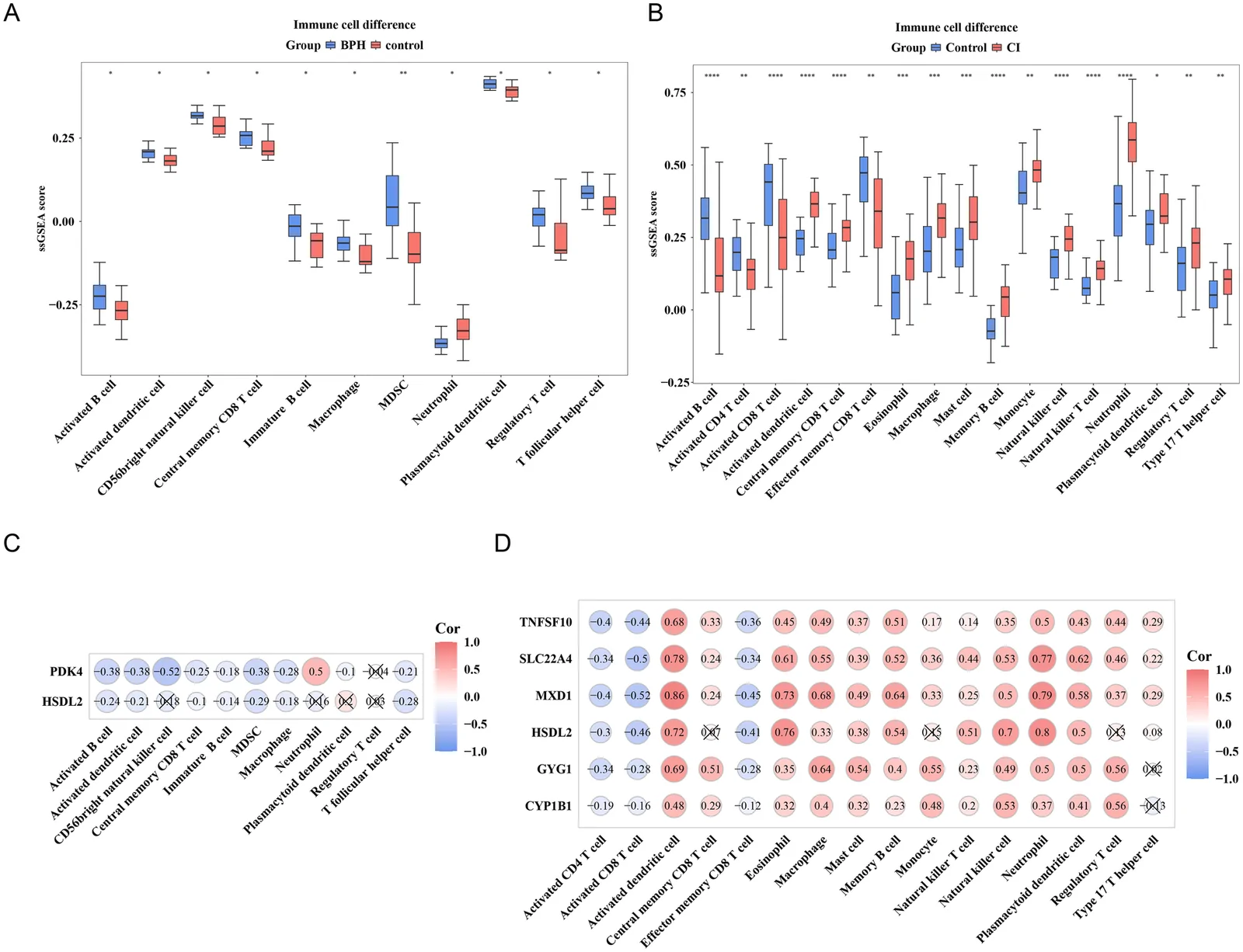
Immune-microenvironment differences between BPH and CI. **(A)** Comparison of immune-cell infiltration between BPH and healthy prostate tissue. Eleven immune-cell types show significant differences (*P* < 0.05); neutrophils are higher in controls, while 10 other immune-cell populations are enriched in BPH. **(B)** Immune-cell infiltration comparison between CI-patient peripheral blood and healthy controls. Seventeen immune-cell signatures differ significantly (*P* < 0.05); four immune-cell types are decreased in CI, and 13 are increased. *P < 0.05, **P < 0.01, ***P < 0.001, ****P < 0.0001 vs. control. (C-D) Correlation heatmaps between hub-gene expression and differentially infiltrated immune cells. **(C)** BPH: PDK4 correlates negatively with activated dendritic cells (cor = −0.52, *P* < 0.05). **(D)** CI: all six CI-related biomarkers show positive correlations with activated dendritic cells (cor > 0.4, *P* < 0.05). Except HSDL2, seven hub genes are positively correlated with neutrophils (|cor| > 0.3, *P* < 0.05).

### 3.6 RT-qPCR experimental validation

RT-qPCR showed that both HSDL2 and PDK4 mRNA levels were significantly lower in the CIwPH group compared with controls (*P* < 0.05; Figures 6A-B), corroborating bioinformatic predictions. Notably, our RT-qPCR validation was performed exclusively on CIwPH subjects. Independent cohorts of isolated CI and isolated BPH were not included in experimental validation, so we cannot disentangle the individual effects of CI or BPH on HSDL2 and PDK4 expression.

**Figure 6:**
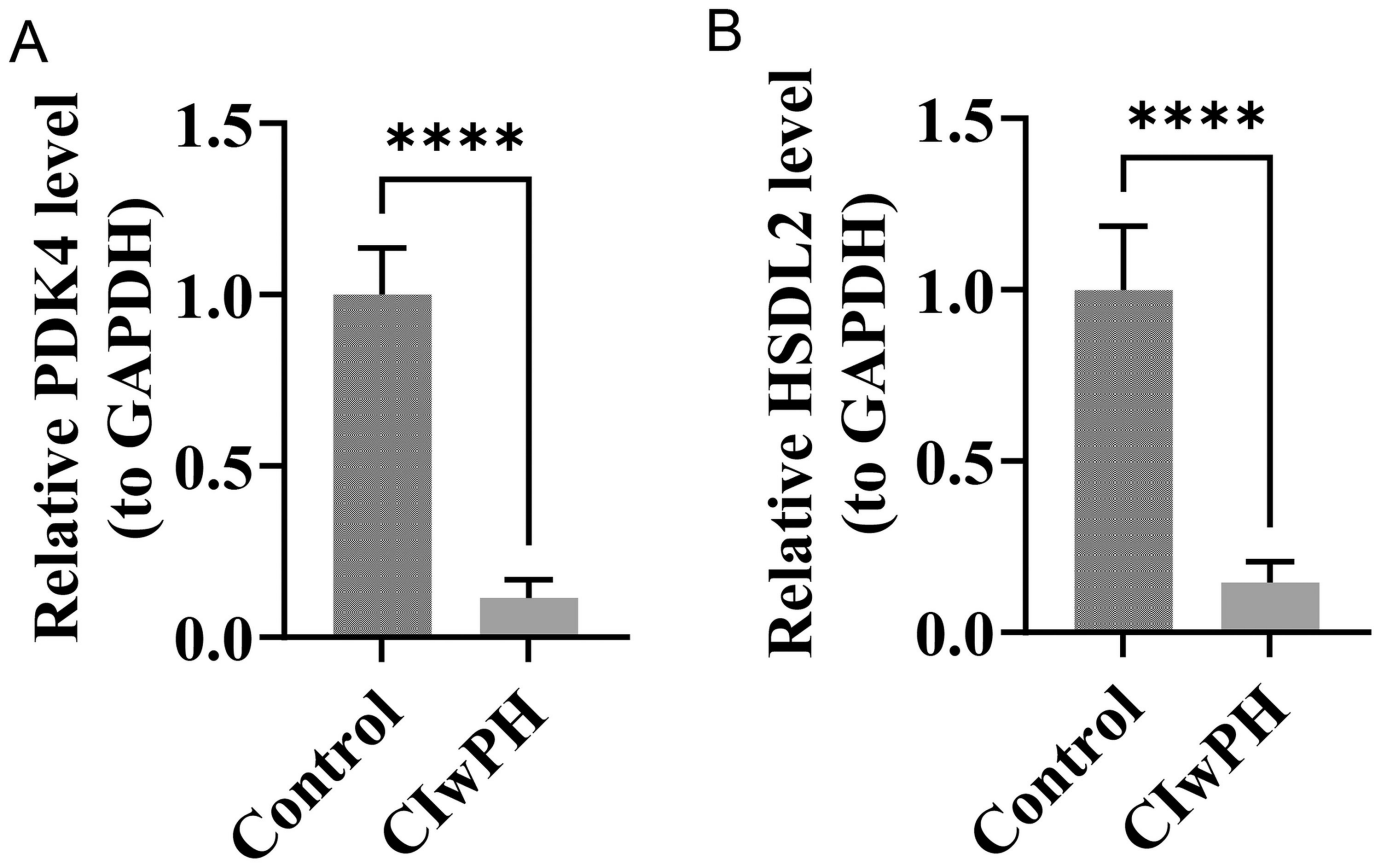
RT-qPCR experimental validation of PDK4 and HSDL2 mRNA expression. **(A)** Relative PDK4 mRNA abundance in control and CIwPH groups. **(B)** Relative HSDL2 mRNA abundance in control and CIwPH groups. Both genes are significantly downregulated in CIwPH. Data are presented as mean ± SEM. *P < 0.05 vs. control.

## 4 Discussion

CI and BPH are common age-related disorders affecting distinct organ systems yet sharing pathophysiological hallmarks: mitochondrial dysfunction, oxidative stress, and dysregulated apoptosis or proliferation (28, 29). With population aging, their comorbidity is rising, yet common molecular mechanisms remain poorly understood. Dissecting shared mitochondrial regulatory networks may open new translational avenues. In this work, integrative bioinformatics plus experimental validation identified eight hub mitochondrial-related biomarkers. HSDL2 and PDK4 were downregulated in BPH and displayed strong diagnostic performance across training and validation datasets. Six genes (CYP1B1, GYG1, HSDL2, MXD1, SLC22A4, TNFSF10) were upregulated in CI with high discriminatory power. Nomogram risk models built on these genes achieved favorable predictive accuracy, suggesting potential utility as adjunct biomarkers complementing existing clinical assessments such as IPSS scoring for BPH and imaging for CI (30). Our identified hub mitochondria-related genes mainly serve as peripheral systemic biomarker candidates for CI and BPH, especially for elderly men with comorbid CI-BPH. They reflect systemic mitochondrial-associated molecular perturbations under disease status, rather than identical pathological alterations occurring *in situ* within prostate and brain tissues.

Functional enrichment linked candidate genes to fatty-acid metabolism, peroxisome function, and apoptotic signaling, all tightly coupled to mitochondrial homeostasis. Impaired fatty-acid oxidation drives lipotoxic neuronal death in cerebral ischemia-reperfusion (31). Androgen-driven lipid reprogramming and metabolic-syndrome-associated metabolic alterations also support stromal-cell proliferation in BPH (32). Peroxisomal dysfunction amplifies ROS accumulation and neuronal injury after ischemic stroke (33). Our observations support the concept that mitochondrial dysregulation acts as a convergence node linking metabolic reprogramming to pathogenesis in both degenerative and hyperplastic age-related diseases.

HSDL2 and PDK4 emerged as shared MRGs. HSDL2 participates in cholesterol homeostasis and metabolic-stress responses (34–36). PDK4 inhibits pyruvate-dehydrogenase complex and modulates glucose metabolism, and has been implicated in ischemic stroke and BPH progression (37, 38). High-glucose conditions repress PDK4 in prostatic epithelial cells, shifting metabolism toward aerobic glycolysis and promoting proliferation and epithelial-mesenchymal transition, reminiscent of Warburg-type metabolic reprogramming in hyperplastic tissues. Downregulation of HSDL2 and PDK4 in BPH and in CIwPH patients may represent a compensatory response to mitochondrial stress and metabolic remodeling. Under ischemia, reduced PDK4 activity may initially support glycolytic ATP generation under hypoxia, yet sustained suppression of pyruvate-dehydrogenase-dependent oxidative phosphorylation eventually worsens neuronal energy failure (39). HSDL2 downregulation in BPH may reflect androgen-driven perturbation of prostatic cholesterol homeostasis and deserves further mechanistic investigation. In isolated cerebral infarction, up-regulated HSDL2 may represent an adaptive stress response triggered by brain ischemia, participating in cholesterol and lipid homeostasis to counteract ischemic oxidative injury. However, under the dual-disease burden of concurrent CI and BPH, combined metabolic disturbance from prostatic hyperplasia and cerebral ischemia may jointly repress HSDL2 transcription. Clinically, this reversed expression pattern suggests that the biomarker performance of HSDL2 cannot be simply extrapolated from single-disease cohorts to patients with comorbid CI-BPH; comorbidity-specific molecular alterations should be considered when applying this gene as a molecular marker in elderly male populations.

In CI, upregulation of CYP1B1, GYG1, MXD1, SLC22A4, and TNFSF10 aligns with known roles in oxidative stress, inflammation, and apoptosis. CYP1B1 modulates ACSL4-dependent lipid peroxidation and ferroptosis in ischemic brain injury via PKC-FBXO10 signaling, possibly representing an endogenous neuroprotective response whose net effect is context-dependent (40). TNFSF10 mediates apoptosis in neurons and prostate cells (41–45). GYG1 encodes glycogenin-1 and reflects glycogen-metabolism rewiring under ischemic energy depletion (46). MXD1 is a MYC-target transcriptional repressor linked to cell-cycle arrest and apoptosis under hypoxia (47). SLC22A4 is an organic-cation transporter expressed in vascular and immune compartments that may shape post-ischemic neuroinflammation across the blood-brain barrier (48). Collectively, these MRGs contribute to CI pathogenesis through interconnected metabolic-stress, immune-regulatory, and programmed-cell-death pathways.

Immune-infiltration analyses revealed divergent microenvironmental remodeling. Activated B-cell and neutrophil infiltration trends were opposite in BPH versus CI. In BPH, multiple immune-cell subsets are enriched within hyperplastic tissue, consistent with chronic inflammation driving stromal proliferation via cytokines such as IL-8 and TGF-*β* (49). Reduced neutrophil abundance in BPH lesions relative to controls may reflect dampened innate immune surveillance with disease progression. In CI peripheral blood, decreased activated B-cell and certain T-cell signatures correspond to systemic immune exhaustion after acute ischemic injury, whereas elevated neutrophil levels promote blood-brain-barrier disruption and secondary neuronal damage (1). Hub-gene expression correlated with dendritic-cell and neutrophil signatures. All six CI biomarkers correlated positively with activated dendritic cells, suggesting mitochondrial-gene-driven metabolic signals may modulate dendritic-cell activation. In BPH, negative correlation between PDK4 and activated dendritic cells points toward distinct local immunosuppressive mechanisms mediated by metabolic reprogramming (38). These findings support the emerging paradigm that mitochondrial dysfunction shapes immune responses in both degenerative and proliferative disease. Regarding the confounding effects caused by different sample sources, we believe that the immune cell profiles obtained from solid prostate tissue (BPH) and peripheral blood circulation (CI) are fundamentally incomparable. The opposite infiltration trends of activated B cells and neutrophils may be partly attributed to the tissue-specific immune landscape rather than purely disease-specific pathological changes. When directly comparing the immune microenvironment cross-diseases between these two datasets, one must interpret with extreme caution.

RT-qPCR confirmed significant downregulation of HSDL2 and PDK4 in CIwPH patients, validating bioinformatic observations in BPH datasets and supporting their biomarker potential for comorbid cohorts. Of note, HSDL2 is upregulated in isolated CI but downregulated in CIwPH, implying distinct metabolic adaptations under dual-disease burden that merit further mechanistic study. Previous studies have also shown that the disruption of mitochondrial division and fusion homeostasis can drive excessive proliferation of stromal cells in BPH (50). We further linked this mechanism to the findings of the MRG study: Although the current gene panels mainly focus on mitochondrial metabolism and apoptosis-related characteristics, the imbalance of division and fusion plays a role as an upstream driving factor for mitochondrial dysfunction in BPH; integrating genes related to division and fusion will become an important direction for our subsequent research.

Several limitations should be acknowledged. First, public-dataset sample sizes are relatively small and patient heterogeneity may limit generalizability. Second, mechanistic validation in cellular and animal models is required to define causal roles for these hub genes. Third, gene-immune-cell correlations are observational; functional causality remains to be proven. Fourth, our RT-qPCR validation only enrolled CIwPH patients; future studies should include separate cohorts of isolated CI and isolated BPH to fully disentangle disease-specific expression dynamics of HSDL2 and PDK4. Fifth, only two out of eight hub genes (HSDL2 and PDK4) received RT qPCR experimental validation; CYP1B1, GYG1, IFI27, MXD1, SLC22A4 and TNFSF10 remain bioinformatically predicted biomarkers without wet lab experimental confirmation in our clinical samples. Finally, our cross-organ comparison aims to identify shared circulating/systemic molecular features in patients with two age-related diseases, rather than to identify identical local tissue pathogenic pathways in the prostate and brain parenchyma. Cross-tissue transcriptomic comparisons inherently suffer from biases arising from differences in tissue origin.

For CI, blood-derived biomarkers are clinically convenient for minimally-invasive detection, even though they cannot mirror intracellular events inside brain lesions. Future animal-model and cell-based experiments are needed to dissect tissue-specific roles of these hub genes in prostate and brain tissues separately.

## 5 Conclusions

We identified a panel of mitochondria-related genes with diagnostic and immunomodulatory relevance for cerebral infarction and benign prostatic hyperplasia. These findings lay a foundation for developing mitochondria-targeted and immune-modulating therapeutic strategies for multimorbid age-related patients. However, our current study is mainly bioinformatics driven exploratory work, and further pre-clinical and clinical studies are required before therapeutic translation.

## Data Availability

The original contributions presented in the study are included in the article/Supplementary Material, further inquiries can be directed to the corresponding authors.
